# K-Means Clustering-Based Safety System in Large-Scale Industrial Site Using Industrial Wireless Sensor Networks

**DOI:** 10.3390/s22082897

**Published:** 2022-04-09

**Authors:** Dongyeong Seo, Sangdae Kim, Seungmin Oh, Sang-Ha Kim

**Affiliations:** 1 Department of Computer Science and Engineering, Chungnam National University, Daejeon 34134, Korea; dyseo.tm@gmail.com; 2 Medical Infromation Technology Engineering, Soonchunhyang University, Asan 31538, Korea; sdkim.mie@sch.ac.kr; 3 Division of Computer Science and Engineering, Kongju National University, Cheonan 31080, Korea; smoh@kongju.ac.kr

**Keywords:** onsite safety system, k-means clustering, multicasting, local flooding, industrial wireless sensor networks (IWSNs)

## Abstract

A large number of workers and heavy equipment are used in most industrial sizes, and the prevention of safety accidents is one of the most important issues. Therefore, although a number of systems have been proposed to prevent accidents, existing studies assume that workers are gathered in some areas. These assumptions are not suitable for large-scale industrial sites in which workers form as a group and work in a large area. In other words, in a large-scale industrial site, existing schemes are unsuitable for the timely notifying of warnings of threats, and excessive energy is consumed. Therefore, we propose a k-means clustering-based safety system for a large-scale industrial site. In the proposed scheme, workers deployed over a large area are divided into an appropriate number of groups, and threat notification is delivered by a multicasting tree toward each cluster. The notification to workers is delivered through local flooding in each cluster. The simulation results show that the system is able to deliver the notification within a valid time, and it is energy efficient compared to the existing scheme.

## 1. Introduction

Industrial wireless sensor networks (IWSNs) have been exploited to improve productivity and financial profits in various industrial fields, such as manufacturing, construction, agriculture, quarrying, timber harvesting, and so on [1,2,3]. In these industrial sites, since a large number of workers and heavy equipment are being devoted to performing their respective roles, much care is required to prevent safety accidents [4,5]. However, despite paying much attention, safety accidents occur for a variety of reasons. In particular, unexploited industrial sites, such as mining, logging, and drilling, are more difficult to secure than general industrial sites, such as manufacturing factories and farming. For example, the workers are struck by mobile equipment even when the workers wear high-visibility clothing, as required by existing safety codes and standards [6]. Moreover, workers become more vulnerable to threats because they are insensitive to surrounding threats by wearing clothes such as earplugs, goggles, and helmets for safety, paradoxically. To prevent these safety accidents, many studies [7,8,9,10,11,12,13] for safety notification exploiting wireless sensor networks have been proposed, including local flooding, multicasting, virtual storage, etc. However, since the existing schemes did not consider that workers in industrial environments were divided into several groups, the transmission–success ratio is low and energy consumption is high.

Therefore, we design a k-means clustering-based safety system to notify warnings in a timely manner. We assume the following environment for the proposed scheme. First, the sensor nodes for detecting threats are evenly distributed in the industrial site. Second, workers perform their roles by grouping, and there might be some positional changes during the task. In addition, when the task is completed, they move to another location for another task. In this case, the workers report their new position. Third, it is assumed that the workers have a receiver capable of receiving notifications transmitted from the sensor.

To achieve real-time notification delivery, the designed system consists of the following four steps:

1 The workers periodically report their current location through the sensors around them to the sink node, and report separately if there is a change in location when the task is completed. The location reporting period might vary depending on the using equipment in the site.
  (1) If a collision between the mobile equipments and workers is expected, a notification of the threat is immediately send to the relevant workers;
  (2) In the generic case, the sink divides the workers into the appropriate number of clusters based on the locations of the reported workers by exploiting k-means clustering. The information of each worker group is expressed as a center point and a radius;
2 The sink node propagates the clustering information to the whole network to deliver the position of workers to each sensor;
3 The source node that sensed threats such as gas leaks and wildfires constructs a real-time multicasting tree using the received cluster information;
4 When the notification arrives at any node inside the cluster along the multicasting tree, the node performs that local flooding via branch points to deliver the notification to workers located inside the cluster.

Through these steps, threats are delivered to workers within a valid time, and could significantly reduce energy consumption compared to existing schemes. The main contributions of our work in this paper could be summarized as follows:

1 We propose a safety system in large-scale industrial site using k-means clustering;
2 The proposed system consists of three phases: clustering, multicasting, and local flooding;
3 The system can deliver notifications about threats to the worker within a valid time and is more energy-efficient than the existing method;
4 We provide simulation results on the proposed scheme and compare them with [7,14] using several evaluation factors.

The remainder of this paper is organized as follows. In Section 2, we describe the existing notification schemes. In Section 3, we explain the designed system for safety systems consisting of (1) k-means clustering-based worker clustering, (2) multicasting tree construction, and (3) local flooding for real-time notification transfer. The performance evaluation results by the simulation are provided in Section 4. Finally, the proposed scheme and simulation results are summarized in Section 5.

## 2. Related Works

In this section, we briefly summarize the existing schemes based on the local flooding, multicasting, and virtual storage for notifications.

The local flooding scheme is a proposed scheme that aims to reduce the energy waste of flooding, delivering data to the whole network, and is a technique that performs flooding only within any range specified by the user or application. The notification scheme exploiting local flooding consists of two steps: member information collection and notification to members. In the process of information collection, a cluster representing members is created and the center point and radius of the cluster are reported. After the information collection process, Mobile Geocasting (M-Geocasting) [7] transmits notification packets to be flooded into the cluster. In M-Geocasting, the packet is sent toward the center point of the cluster. The local flooding technique can transfer notifications even if the location of members is unclear. However, when a small number of members are widely distributed, local flooding causes unnecessary energy waste. The work in [12] also exploits local flooding similar to M-Geocasting, and prevents collisions and interference during the local flooding process. However, due to the limitation of the local flooding technique, [12] also suffers from a problem similar to M-Geocasting.

The virtual tube scheme is a scheme that delivers data to a user based on the prediction of a movement of the user. The sensor that detects the events receives the movement information of the user from the sink and places the data at the sensors around the location, through which, the user passes in consideration of the movement direction and speed of the user. The work in [8] puts the notification in a virtual rectangular area passing the center point of the cluster. The member workers could passively obtain the notification when encountering the area. Similarly, the virtual tube storage scheme (VTS) [11] exploits virtual tube storage to notify alerts to workers. However, since VTS is also a scheme in which the member manually acquires the notification, it is difficult to receive the notification of the alert within a valid time. In addition, some workers might not be notified within a valid time because the mobility of workers and equipment is not considered.

Multicasting is a method in which a source that detects an event constructs a multicast tree for delivering data to each destination and exploits the tree to deliver data. This scheme usually designs a tree in the directions where the sum of the costs of transmitting data to each destination is the lowest. Multicasting [9,10,13] also collects the location information of equipment and workers and constructs a multicasting tree based on the collected information. However, the construction of a multicasting tree commonly requires a large cost and amount of time. In addition, interference between paths could occur in the transmission to an adjacent destination. Therefore, the notification transmission scheme exploiting the multicasting tree is not suitable for applications that should be accurately transmitted within a valid time.

## 3. K-Means Clustering-Based Safety System for Large-Scale Industrial Site

In this section, we describe the k-means clustering-based safety system for preventing accidents in industrial sites. Since the k-means clustering is an algorithm based on the distance between each point (worker), it is suitable for classification according to the location of the workers. To prevent the accident, the notification about various threats should be delivered to the workers within a valid time. Our main idea is to divide the scattered workers into the appropriate number of clusters. By dividing workers into clusters, real-time notifications exploiting multicasting trees are possible and the energy consumption can be reduced.

This section is divided into four parts. In Section 3.1, we describe the operation overview of a proposed safety system. In Section 3.2, we explain the operation process of the k-means clustering scheme and how to divide scattered workers into the appropriate number of clusters based on the k-means clustering scheme. The multicasting tree construction for each cluster and local flooding within the cluster are described in Section 3.3 and Section 3.3.3, respectively.

### 3.1. Overview

Through this section, we show the overall operation process of the proposed system. First, we assume that all sensor nodes are aware of their own location by GPS [15] or other techniques [16], and know the location of the sink node through interest flooding [17] of the sink node. In addition, each node keeps the location information of its one-hop neighbor nodes by beaconing.

**Figure 1:**
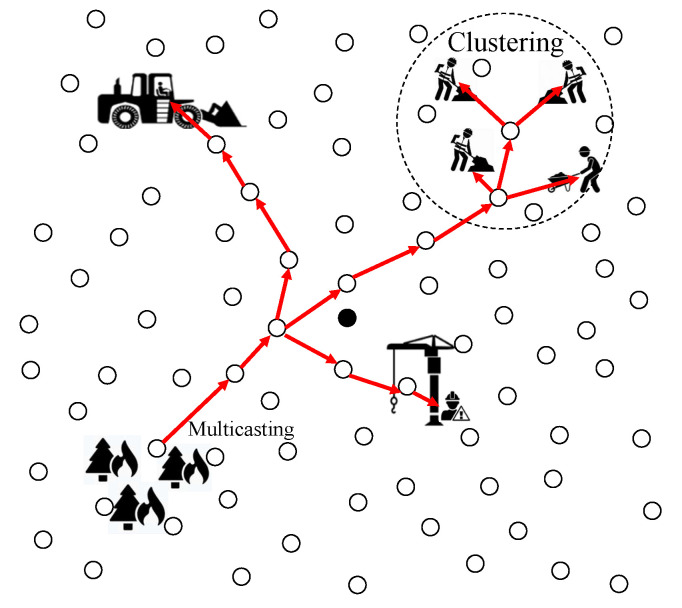
An overview of proposed system.

As shown in Figure 1, the sink node is located at the center of the network, and several workers and equipment are distributed throughout the network to perform their own tasks. Initially, the sink node collects workers’ location information to make decisions and notifications about threats about conflicts between workers. Through the information, the sink node identifies the possibility of accidents, such as collisions between the equipments and workers due to their negligence. If an accident is predicted, the sink immediately notifies the relevant equipments and workers.

After performing notifications for potential threats, the sink node divides workers into the appropriate number of groups based on the k-means clustering scheme. In the clustering process, the sink node can calculate the center point and radius of the cluster with the least average standard error based on the location of the workers. The sink node periodically notifies the information of calculated clusters to the whole network. To increase the lifetime of the network, a cluster head node representing each cluster is selected, such as in [18]. Through the information, the sensor nodes that have detected natural disasters, such as gas leaks and wildfires, construct multicasting trees to alert the threats. First, the sensor node finds the farthest cluster to calculate the reference delivery speed for real-time transmission. Once the reference delivery speed is determined, the sensor node constructs the multicasting tree toward the clusters. If some paths of the tree are longer than the farthest distance initially found, the packet cannot be delivered within a valid time. In this case, the sensor partially modifies the paths in order for the packet to arrive at all clusters in a valid time. Finally, when the packet is delivered in the cluster, the packet is transferred to all workers by regional flooding via branch points.

**Figure 2:**
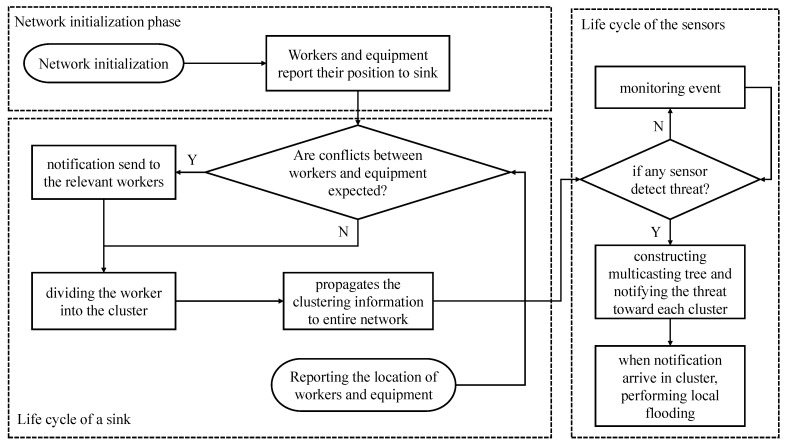
A flowchart of operation process of proposed system.

Figure 2 shows the operation process of the proposed system. After initialization of the network, the sink node repeats the following life cycle:

1 The sink node confirms the possibility of conflict between workers and mobile equipment during the network initialization phase;
  (1) If a possibility of collision is detected, the sink node immediately delivers a notification to the relevant workers;
  (2) After the notification is delivered or if there is no possibility of collision, the clustering is performed;
2 The location information of the workers is propagated throughout the network;
3 If a location report of workers is received, the life cycle is repeated again from 1.

In the case of the sensor node, the following life cycle is followed:

1 The sensor node is always detecting events;
  (1) Therefore, if no event is found, monitoring is continuously performed;
  (2) If the event is found, these steps are followed;
2 A multicasting tree is constructed based on destination information that is received from the sink. In this case, the tree is constructed in consideration of the time limitation required by the application or user;
3 The notification is delivered along the tree, and when the notification enters the cluster, local flooding is performed to notify the threats to workers located inside the cluster;
4 The sensor that has completed the notification performs event detection again. If the sensor detects an event, the life cycle resumes at 1.

### 3.2. Worker Clustering

In our proposed system, after performing threat notifications about conflicts between workers, the sink node performs clustering for real-time multicasting. Thus, we describe how to divide workers and equipment into the appropriate number of clusters by the k-means clustering scheme in this section.

**Figure 3:**
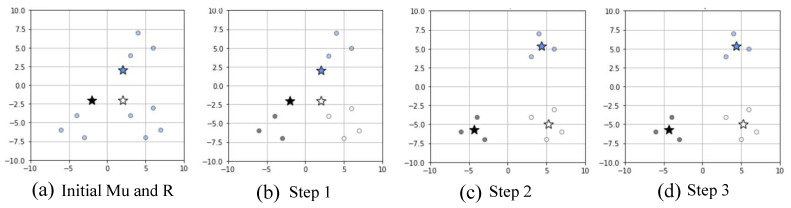
An example of k-means clustering.

Figure 3 shows an example of the k-means clustering scheme. Figure 3a shows the results of arbitrarily initializing the initial center vector μ and indicator variable *R*, respectively, when the number of clustering is three. Figure 3b–d are the results of performing the k-means clustering algorithm once, twice, and thrice, respectively. The k-means algorithm iterates until μ and *R* are no longer changed. In Figure 3, there is no difference between step 3 (Figure 3d) and step 2 (Figure 3c), the clustering algorithm determines that optimal clustering has been performed, and the algorithm is terminated.

**Figure 4:**
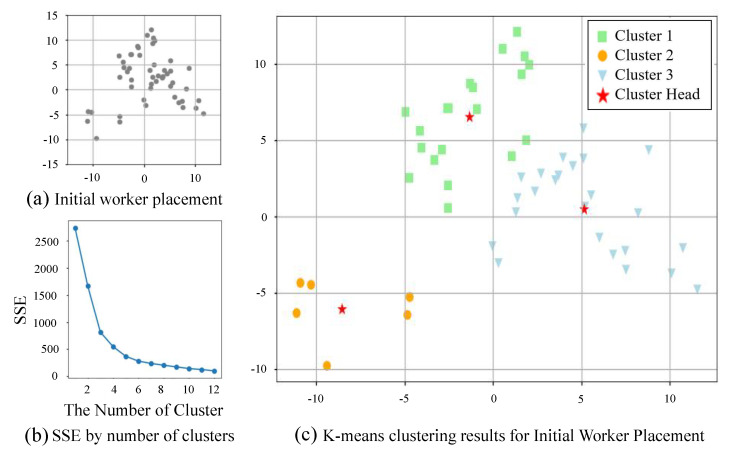
An example of elbow method.

As inferred from the above clustering process, the number of clusters is one of the important factors in the clustering process. If there were two or four clusters, the results would not have been as shown in Figure 3d. Thus, we exploit the elbow method, as shown in Figure 4, to find the appropriate number of clusters about scattered workers. The elbow method is used to compare calculated sum of squares error (SSE) values by increasing the number of clusters from one to n. The rapidly decreasing point of SSE values corresponds to the elbow, which is the optimal number of clusters. In the case of the elbow method, the SSE decreases rapidly every time the number of clusters increases, which decreases very slowly when an appropriate number of clusters is found. We determined that the section in which the difference between the previous SSE value and the current SSE value is significantly reduced is the appropriate number of clusters. Figure 4b shows that, as a result of the application of the elbow method for Figure 4a, the reduction ratio decreases sharply when the number of clusters is three. Figure 4c shows that the workers in Figure 4a are divided into three clusters according to the results of the elbow method, and the clusters are properly classified.

Through the above-mentioned process, the sink node could find the appropriate number of clusters to construct a multicasting tree based on the locations of reported workers. However, in a variety of industrial environments, workers could shift their positions according to their roles, in which case, they report their movement information to the sink. If a worker’s movement information is reported to the sink, the sink node identifies the threat to the movement and notifies the threat if it exists. In addition, the sink node performs re-clustering and re-propagates the clustering information to the whole network.

### 3.3. Real-Time Multicasting

In this section, we describe a multicasting scheme to deliver data within a valid time to all workers in the cluster. The method of multicasting is largely divided into three stages: (1) calculation of the longest distance, (2) multicasting tree construction, and (3) local flooding via branch points inside the cluster. First, the source node calculates the distance to the farthest cluster to find the reference delivery speed for real-time transmission. Subsequently, the source node constructs a multicasting tree oriented toward the center of each cluster. This multicasting tree focuses on reducing the number of hops, with the shortest distances from the source to the center of each cluster. Finally, when the packet arrives in the cluster, the sensor node that received it performs local flooding via branch points inside the cluster, allowing all workers to receive the packet.

#### 3.3.1. Calculation Longest Distance for Finding Reference Delivery Speed

We calculate the reference delivery speed based on a spatiotemporal approach [19]. The reference speed is calculated according to the required time and distance. Therefore, we explain a scheme for calculating the longest distance to find the reference delivery speed in this section.

To satisfy the real-time requirement of the application, the reference delivery speed should be satisfied at all nodes. That is, when transmitting the packet to the next node, all next nodes should select a node capable of relaying the packet faster than the reference delivery speed in order to meet the requirement. In the process, the temporal requirement is determined according to the application, whereas the spatial requirement is defined as the Euclidean distance between the source and the destination. To calculate the Euclidean distance, we assume that each node knows its location by GPS or any localization algorithms. However, the end-to-end distance between a source node and workers cannot be defined, as the data delivery towards each worker is based on local flooding within the area.

**Figure 5:**
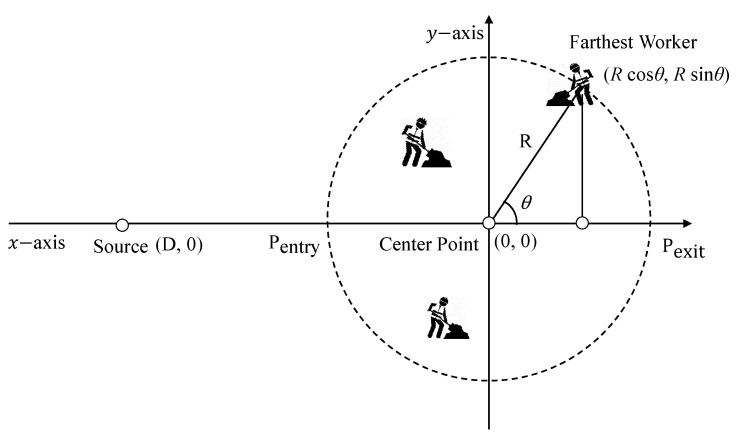
Calculation of longest distance in the cluster.

Therefore, a source node defines the distance and calculates the reference delivery speed after obtaining the location information of the furthest cluster [7,8,12]. In order to derive the delivery speed for every worker in a cluster, we consider the maximum (longest) distance in the movable area. In the area, as the farthest point from the entry point might be located on the ring, we calculate the distance between two points on the ring. In Figure 5, we assume that the center point and the source node are on the coordinates (0,0) and (D,0), respectively. Each point on the circle can be represented with the angle *θ*: (*Rcos θ*, *Rsin θ*). The distance to each point is presented as follows:

(1) f(θ)=(D+Rcosθ)+|Rsinθ|.

Through the differential equation, from Equation (1), we can obtain the farthest points on the circle: (θ=3/4π or 5/4π) as $f^{\prime }\left(3/4\pi \right)$ = $f^{\prime }\left(5/4\pi \right)$ = 0 and $f^{\prime \prime }\left(3/4\pi \right)<0,f^{\prime \prime }\left(5/4\pi \right)<0$. Therefore, the maximum distance is $\left(D+\sqrt{2}R\right)$. With the maximum distance, the source node makes the reference delivery speed, which will be maintained during the packet delivery.

#### 3.3.2. Multicasting Tree Construction Based on the Longest Distance

In this section, we describe how to construct a multicasting tree based on the longest distance calculated in Section 3.3.1. The multicasting tree is constructed through a perpendicular-based heuristic method [14]. This scheme consists of the following three phases. First, the source node draws a baseline between the cluster heads. Second, the path to each cluster head diverges vertically from the baseline. Finally, if the path length to each cluster head is longer than the longest distance, the divergence point of the path is shifted.

**Figure 6:**
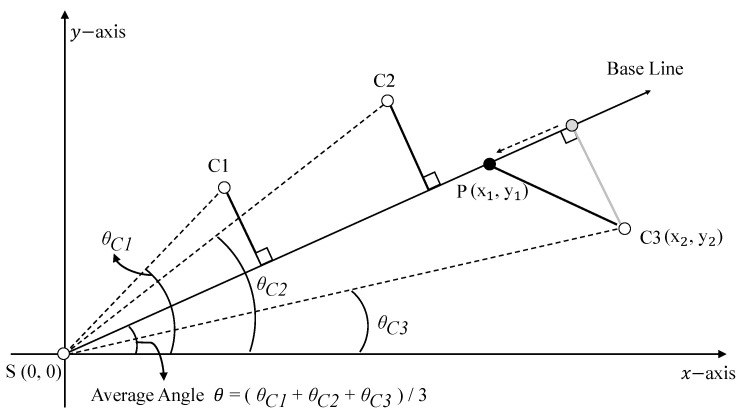
An example of multicasting tree construction.

Figure 6 shows an example of multicasting tree construction. In order to draw the path of the multicasting tree, the average angle of the cluster heads is used in the process of establishing the baseline. In other words, the source node calculates a baseline depending on the average angle of the source node, the cluster head, and the *x*-axis (Figure 6 shows an example of calculating the average angle). After determining the baseline, a perpendicular line is drawn to each cluster head to complete the first multicasting tree. However, this multicasting tree could be longer than the previously calculated longest distance. In this case, the real-time transmission cannot be achieved through the calculated reference delivery speed in Section 3.3.1. Therefore, the branch point should be recalculated. In Figure 6, we assume that the existing path to cluster head C3 is longer than the calculated longest distance. To minimize the length of the new path via a new branch point, the point P should satisfy the notion that the length of S-P-C3 is equal to the longest distance. Thus, $\sqrt{{x_{1}}^{2}+{y_{1}}^{2}}+\sqrt{{\left(x_{2}-x_{1}\right)}^{2}+{\left(y_{2}-y_{1}\right)}^{2}}$ should be the same as the longest distance. In addition, since the point P is above the baseline, it can be calculated.

#### 3.3.3. Real-Time Data Delivery via Branch Points inside Cluster

After a multicasting tree is constructed through the previous section, the packet for the notification is delivered along the tree and enters each cluster. In this section, we describe how to perform local flooding when a packet reaches a cluster.

When a packet has entered a cluster, the point opposite the entry point is defined as the exit point, and the delivery process of the packet from the entry point to the exit point is defined as the main forwarding. In the process of performing the main forwarding, the process of forwarding the packet to the other nodes inside the cluster is defined as branch forwarding. The branch forwarding area is divided according to the wireless transmission range. By separating branch forwarding regions, the energy consumption is reduced by preventing all nodes from participating in communication during packet flooding. There are ⌈2R/r⌉ branching points in the main forwarding in a cluster, whose radius is *R*, and the radio range of the sensor nodes is *r*.

**Figure 7:**
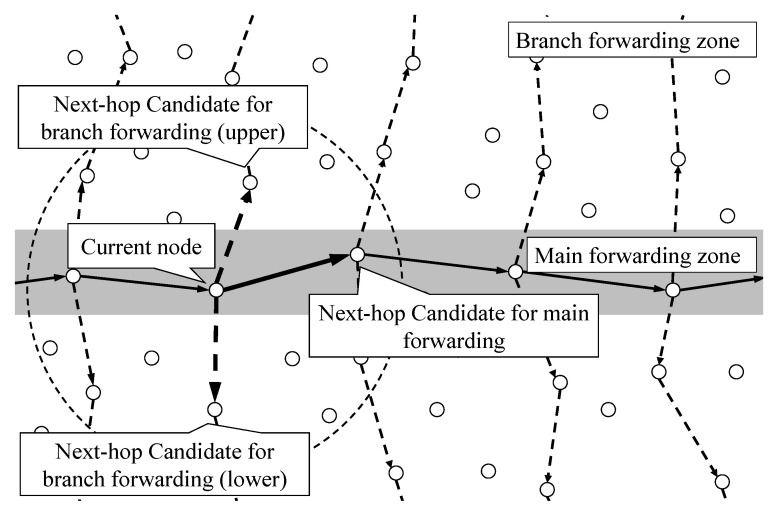
Point selection for main and branch forwarding.

In the process of performing local flooding inside the cluster, three nodes are selected, as shown in Figure 7. One node is for main forwarding, and the other two nodes are for branch forwarding toward each orthogonal direction. However, while relaying the packet, collision and interference could occur due to the simultaneous transmission of several sensors within the radio range of the nodes that are caching a packet. To alleviate the problem, time-slot-based transmission is required in order to share the opportunity to relay among the branch nodes without collision or interference. In time-slot-based transmission, each slot is assigned to the node that transmits the packet for branching. The node can transmit packets between time intervals between its hop delay to a time deadline. However, controlled relaying is required to prevent each node transmitting packets in the overlapped time slot. For relay control to prevent the overlapped time slot, we first assume that the nodes are time-synchronized using the time synchronization schemes [20,21]. When each next candidate node performs a packet transmission, the node should exploit different time slots. Although the node exploits a different time slot, packet collision and interference could occur because the packet on the node is independently relayed. To prevent this potential problem, an additional scheduling process to determine the order of transmission is required. The current node divides the time interval into three slots and allocates the next candidate nodes to each time slot. One slot is assigned to the candidate for main forwarding, and the other two slots are assigned to the candidate for branch forwarding. Each candidate node transmits a packet at the assigned time slot, and the time slot allocation conditions are as follows:

- The first time slot is assigned to the node that has the shortest hop delay;
- The last time slot is assigned to the node that has the longest time deadline;
- The remaining time slot is assigned to the node that has not been assigned in any time slot.

Through the above conditions, the proposed system could achieve the transmission of notifications about threats to all workers in the cluster without collision and interference.

## 4. Performance Evaluation

In this section, we evaluate the performance of the proposed system. The specification of sensor nodes follows the standard of the wireless highway addressable remote transducer protocol (WirelessHART) [22], which is a wireless sensor networking technology that is widely exploited in industrial sites. To demonstrate the performance when the proposed system transmits packets after dividing workers into the appropriate number of clusters, we compare with M-Geocasting [7], which considers workers as one group, and the multicast protocol for real-time data dissemination (MPRD) [14], which constructs a multicasting tree directed at each worker. The simulation environment settings are as follows. Considering the characteristics of large-scale industrial environments, we assume that 625 sensor nodes are uniformly deployed in a 2500 m × 2500 m square area, with 200 workers performing their work. The workers could work alone or in groups. It is assumed that the worker’s moving speed is 4 km/h according to the average walking speed of a person. There might be small movements while workers are working; however, they are within the communication range of the sensor. Furthermore, we assume that workers could often move according to their work. It is assumed that they would stay at least 3 min after moving.

**Figure 8:**
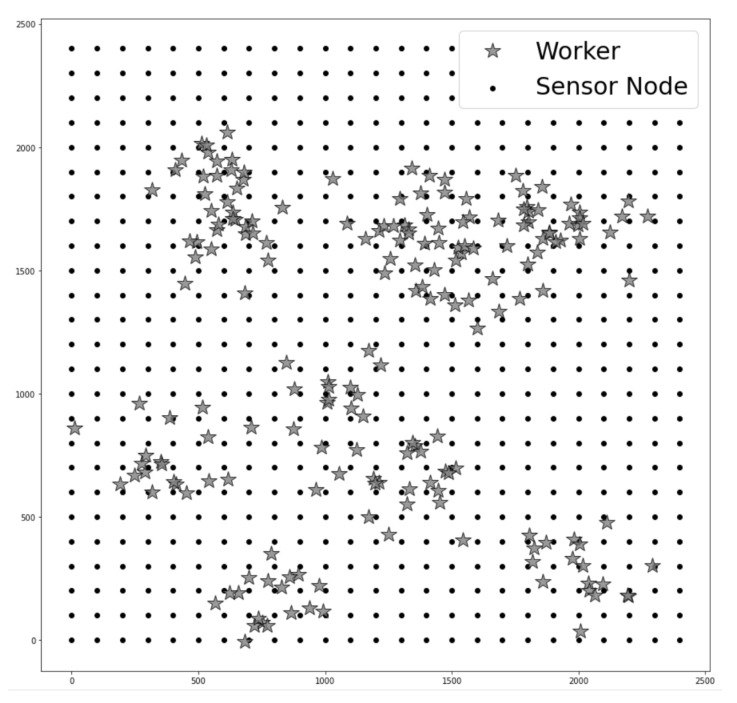
An example of industrial site topology.

Figure 8 is one of the examples of the simulation environments described earlier, and performance evaluations have been executed 20 times in various topologies (changes to the initial location of workers and their mobility). The simulation result in the figures is the average values. The simulation factors and terms for performance evaluation are defined as follows:

- In-time Packet Transmission Success Ratio is defined as the number of messages arriving at the workers in a timely manner divided by the number of messages sent by the source node;
- Energy Consumption is defined as the average energy consumption exploited to transmit and receive packets of sensor nodes participating in data transmission;
- The Number of Workers is defined as the number of workers simultaneously moving in the network;
- Radio Range can be influenced by various industrial environments. Thus, simulations are conducted on various radio ranges of sensor nodes.

### 4.1. Simulation Results for the In-Time Packet Transmission Success Ratio

**Figure 9:**
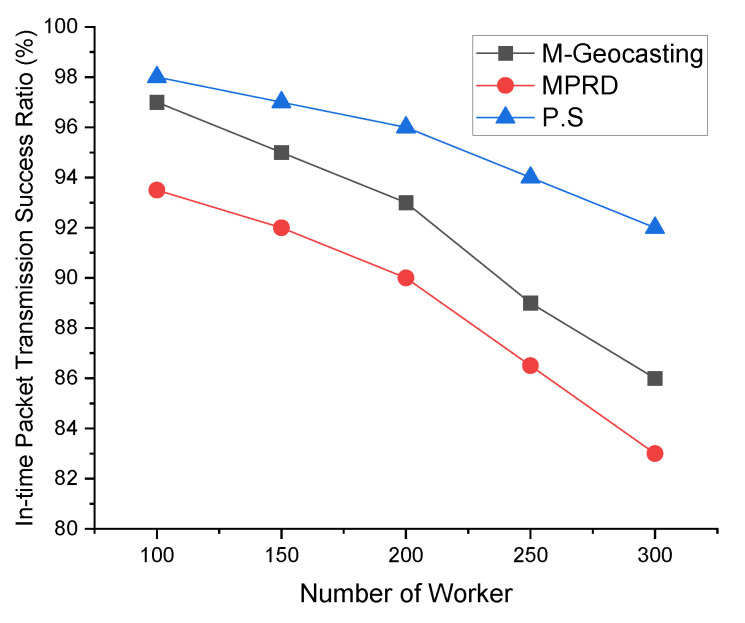
Comparison of in-time packet transmission success ratio according to the number of workers.

Figure 9 shows the in-time packet transmission success ratio according to the number of workers. As the number of workers increases, the average distance between workers or the distribution within the cluster changes, and the distance between workers from the source that detected the event tends to be longer. As a result, in case of M-Geocasting, where all workers are organized into one cluster, the size of the cluster becomes larger. Therefore, it can be seen that the in-time packet transmission success ratio for faraway workers is reduced. In the case of MPRD, the number of branches increases as the number of workers increases, and the probability of collisions and interference occurring between each branch is increased. Therefore, the in-time packet transmission success ratio is decreased because of the transmission failure. In the case of the proposed system, additional scheduling for the transmission of branches is performed while dividing the workers into the appropriate number of clusters through the k-means clustering scheme. Therefore, the in-time packet transmission success ratio of the proposed system is higher than the previous methods, regardless of the number of workers.

**Figure 10:**
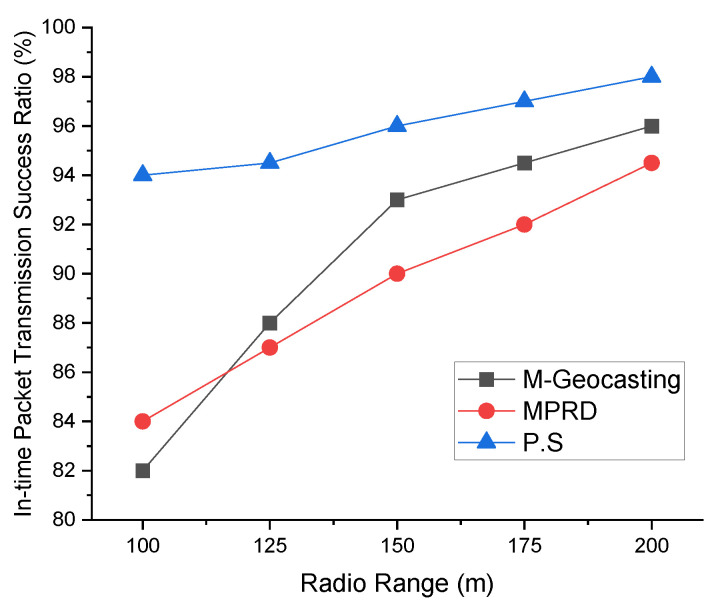
Comparison of in-time packet transmission success ratio according to the radio range.

Figure 10 shows the in-time packet transmission success ratio according to the radio range. When the radio range expands, the transmission hop counts decrease due to the nature of the wireless network transmission by a hop-by-hop manner. In other words, widening the radio range means reducing/increasing the network scale. Therefore, in the case of M-Geocasting, the in-time packet transmission success ratio increases as the radio range increases. On the other hand, since the hop counts increase as the radio range decreases, the in-time packet transmission success ratio decreases. In the case of MPRD, as the radio range increases, the number of workers included in a one-hop radio range is increased. That is, as the number of workers in a one-hop radio range increases, the number of branches decreases. This phenomenon leads to a reduction in collisions and interference between transmission, resulting in a slight increase in the in-time packet transmission success ratio. In the case of the proposed system, the system is not significantly affected by the radio range. However, when the radio range is increased, the in-time packet transmission success ratio is slightly increased for similar reasons to M-Geocasting.

### 4.2. Simulation Results for the Energy Consumption

**Figure 11:**
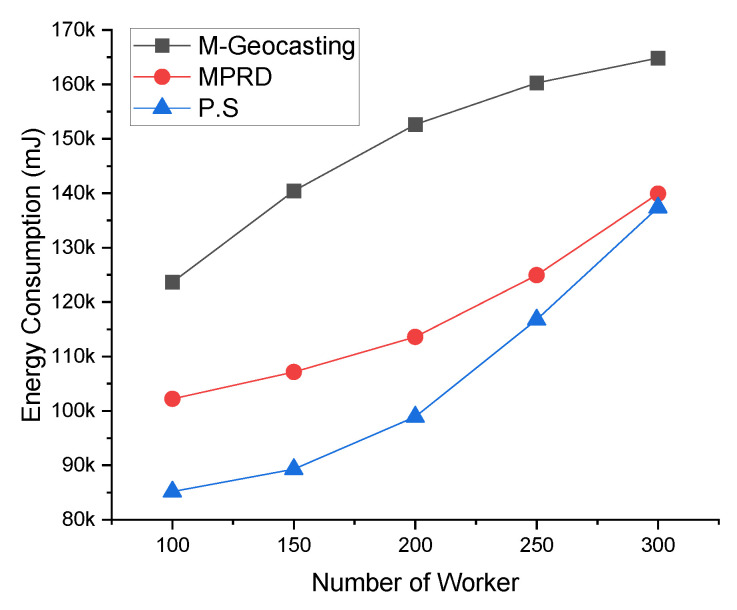
Comparison of energy consumption according to the number of workers.

Figure 11 shows the energy consumption according to the number of workers. Increasing the number of workers generally leads to an increase in the number of destinations, and this phenomenon increases the average energy consumption. However, in the case of M-Geocasting, only the size of the cluster changes as the number of workers changes, because it organizes all workers into one cluster. Therefore, the energy consumption does not increase significantly because the cluster size changes are relatively small as the number of workers increases. In the case of MPRD, as the number of workers increases, the number of branches for packet transmission increases. Therefore, the energy consumption increases in proportion to the increase in the number of workers. In this process, the energy consumption is further increased when considering the retransmission process that recovers the failure of transmission due to the collision and interference. Otherwise, the energy consumption does not increase significantly by the loss packet in the transmission process. For similar reasons to M-Geocasting for the proposed system, increasing the number of workers does not significantly increase the energy consumption (flooding into the cluster is not affected by the number of workers). However, if the number of clusters increases as the number of workers increases, the energy consumption increases because additional flooding is required for the added cluster.

**Figure 12:**
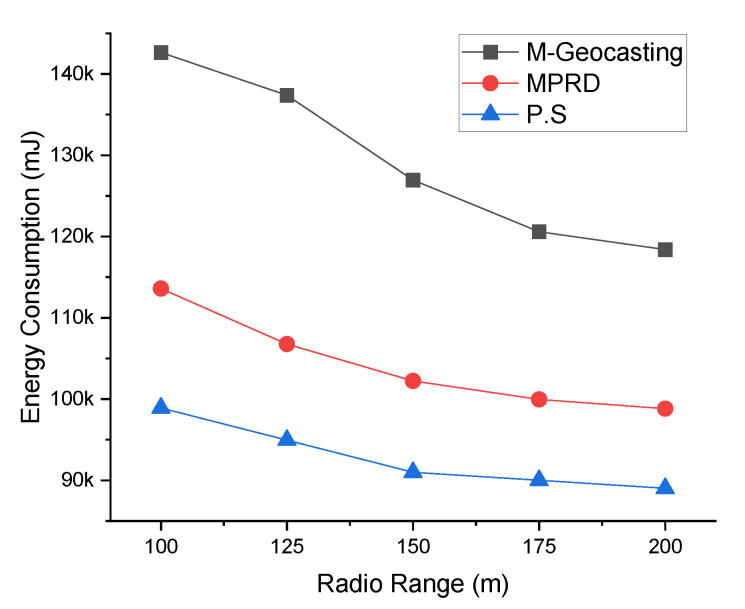
Comparison of energy consumption according to the radio range.

Figure 12 shows the energy consumption according to the radio range. Changes in the radio range engender changes in the hop count, as mentioned above. In the case of M-Geocasting, the size of the cluster is fixed to include all workers. Therefore, as the radio range increases, the packet for the notification could be delivered to all workers in the cluster with fewer transmissions. This phenomenon leads to a reduction in energy consumption. In the case of MPRD, extending the radio range would reduce the number of collisions and interference between transmission and reduce the hop count. Thus, it also leads to a decrease in energy consumption. In the case of the proposed system, similar to the existing schemes, the number of transmissions would decrease, resulting in a slight reduction in energy consumption.

## 5. Conclusions

Although various industries are upgrading today, various safety accidents are occurring for workers. Despite paying a lot of attention to this problem, such as through safety codes and standard legislation, ironically, workers have become more vulnerable to threats. Therefore, appropriate notification of threats to prevent safety accidents is required, and various studies have been proposed. However, since they did not consider the characteristics of workers who are working in an industrial environment, they failed to deliver the notification packet within a valid time and consumed excessive energy.

To deal with these problems, in this paper, we proposed a k-means cluster-based safety system considering the characteristics of clustered workers in an industrial environment. The proposed system consists of the following three steps: (1) dividing the workers into the appropriate number of clusters, (2) constructing a multicasting tree toward each cluster, and (3) local flooding via branch points in the cluster. Through the three steps, the proposed scheme aims to consume less energy than existing schemes, while simultaneously delivering notification packets within a valid time.

We analyzed the performance of the proposed safety system from the aspect of energy consumption and the in-time packet transmission success ratio with computer simulation. The results show that the proposed safety system has a maximum 10% and 14% increase in the in-time packet transmission success ratio after varying the number of workers and radio range, respectively, compared to existing schemes. In addition, the energy consumption of the proposed safety system decreases by 36% and 30% or more, after varying the number of workers and radio ranges, respectively, than the existing method.

The proposed system has a high performance for situations in which workers move intermittently, such as quarrying and timber harvesting. However, for workers who continue to move locations, such as in agriculture, frequent location updates are required, leading to excessively wasting energy. Therefore, further research in terms of worker mobility is required to ensure the safety of workers in various industries.
